# Unscrambling the genomic chaos of osteosarcoma reveals extensive transcript fusion, recurrent rearrangements and frequent novel TP53 aberrations

**DOI:** 10.18632/oncotarget.6567

**Published:** 2015-12-11

**Authors:** Susanne Lorenz, Tale Barøy, Jinchang Sun, Torfinn Nome, Daniel Vodák, Jan-Christian Bryne, Anne-Mari Håkelien, Lynnette Fernandez-Cuesta, Birte Möhlendick, Harald Rieder, Karoly Szuhai, Olga Zaikova, Terje C. Ahlquist, Gard O. S. Thomassen, Rolf I. Skotheim, Ragnhild A. Lothe, Patrick S. Tarpey, Peter Campbell, Adrienne Flanagan, Ola Myklebost, Leonardo A. Meza-Zepeda

**Affiliations:** ^1^ Department of Tumor Biology, Oslo University Hospital, Norwegian Radium Hospital, Oslo, Norway; ^2^ Genomics Core Facility, Department of Core Facilities, Oslo University Hospital, Norwegian Radium Hospital, Oslo, Norway; ^3^ Department of Molecular Oncology, Institute for Cancer research, Oslo University Hospital, Norwegian Radium Hospital, Oslo, Norway; ^4^ Clinic for Cancer, Surgery and Transplantation, Oslo University Hospital, Norwegian Radium Hospital, Oslo, Norway; ^5^ Norwegian Cancer Genomics Consortium, Norway; ^6^ Department of Translational Genomics, Center of Integrated Oncology Cologne–Bonn, University of Cologne, Cologne, Germany; ^7^ Genetic Cancer Susceptibility Group, International Agency for Research on Cancer (IARC-WHO), Lyon, France; ^8^ Institute for Human Genetics, University Hospital Düsseldorf, Düsseldorf, Germany; ^9^ Department of Molecular Cell Biology, Leiden University Medical Center, Leiden, The Netherlands; ^10^ Centre for Cancer Biomedicine, Faculty of Medicine, University of Oslo, Oslo, Norway; ^11^ Wellcome Trust Sanger Institute, Hinxton, UK; ^12^ Royal National Orthopaedic Hospital, Middlesex, UK; ^13^ UCL Cancer Institute, University College London, London, UK; ^14^ Institute for Clinical Medicine, Faculty of Medicine, University of Oslo, Oslo, Norway

**Keywords:** bone cancer, osteosarcomas, gene fusion, trans-splicing, DNA repair

## Abstract

In contrast to many other sarcoma subtypes, the chaotic karyotypes of osteosarcoma have precluded the identification of pathognomonic translocations. We here report hundreds of genomic rearrangements in osteosarcoma cell lines, showing clear characteristics of microhomology-mediated break-induced replication (MMBIR) and end-joining repair (MMEJ) mechanisms. However, at RNA level, the majority of the fused transcripts did not correspond to genomic rearrangements, suggesting the involvement of trans-splicing, which was further supported by typical trans-splicing characteristics. By combining genomic and transcriptomic analysis, certain recurrent rearrangements were identified and further validated in patient biopsies, including a *PMP22-ELOVL5* gene fusion, genomic structural variations affecting *RB1, MTAP/CDKN2A* and *MDM2*, and, most frequently, rearrangements involving *TP53.* Most cell lines (7/11) and a large fraction of tumor samples (10/25) showed *TP53* rearrangements, in addition to somatic point mutations (6 patient samples, 1 cell line) and *MDM2* amplifications (2 patient samples, 2 cell lines). The resulting inactivation of p53 was demonstrated by a deficiency of the radiation-induced DNA damage response. Thus, *TP53* rearrangements are the major mechanism of p53 inactivation in osteosarcoma. Together with active MMBIR and MMEJ, this inactivation probably contributes to the exceptional chromosomal instability in these tumors. Although rampant rearrangements appear to be a phenotype of osteosarcomas, we demonstrate that among the huge number of probable passenger rearrangements, specific recurrent, possibly oncogenic, events are present. For the first time the genomic chaos of osteosarcoma is characterized so thoroughly and delivered new insights in mechanisms involved in osteosarcoma development and may contribute to new diagnostic and therapeutic strategies.

## INTRODUCTION

The chaotic chromosomes and heterogeneity of osteosarcomas has made the identification of pathognomonic mutations difficult. Spectral karyotyping has revealed a large number of structural and chromosomal aberrations, with translocations generating complex derivative chromosomes apparently harboring large numbers of fusion sequences [1]. The complexity is exacerbated by exceptionally high frequency of copy number changes [2]. Due to the hyperdiploid constitution of many different derivatives, with hidden translocations, amplified segments and deletions, as well as double minute chromosomes, most cell divisions would be expected to give daughter cells with different karyotypes, resulting in high intratumor heterogeneity. In spite of this, core components of the scrambled genome will be selected for and may be identified by being present in the majority of cells, because over time many of the variant genomes will be lost from the population. Although cell lines have over many years been selected for *in vitro* growth properties, they are important workhorses of preclinical research, and maintain many of the central oncogenic mechanisms [3]. Observations initially identified in cell lines may subsequently be validated in patient samples, as has been done here. A clear advantage of the availability of cell line data is that *in vitro* models for functional analysis are immediately available.

An increasing number of pathognomonic translocations have been identified in sarcoma subtypes, but not yet in osteosarcomas. On the other hand, focused copy number changes could be identified, among them, frequent deletion of *LSAMP* [4, 5], and amplification of *CDK4* and *MDM2* [6], leading to inactivation of *RB1* [7] and *TP53* [8], respectively. Especially the RB1 and p53 pathways appear to be important for osteosarcoma development, as survivors from retinoblastoma have a high risk of secondary osteosarcomas [9], and sarcomas are prevalent in Li-Fraumeni families with germ line mutations of *TP53* [10]. However, it was for a long time an enigma why mutations in *TP53* had only been observed in about 15-20 % of sporadic osteosarcomas [11, 12], although p53 is inactivated in an additional 10-20 % by amplification and overexpression of *MDM2* [13-15]. Some complementary mechanisms have been proposed [16], but only recently next generation sequencing (NGS) analysis revealed frequent aberrations of *TP53* in osteosarcoma by genomic rearrangements that would be missed by traditional mutation analysis [17, 18]. Also focused but random *kataegis* was observed, but not affecting the *TP53* region [19]. However, in these study only clinical samples were interrogated, and no functional studies on the effect of *TP53* rearrangements could be done.

## RESULTS

### RNA sequencing and identification of fusion transcripts in cell lines

Fusion transcripts were identified by sequencing the transcriptomes of 11 osteosarcoma cell lines (see Supplementary Table S1 for an overview over all samples used). On average, 40 million paired-end reads per sample were generated. A total of 502 candidate fusion transcripts were detected after filtering, varying from a few candidates to more than a hundred fusions per sample (Supplementary Table S2).

Seventeen candidate fusions found in multiple samples and/or showing intact exon structure were chosen for validation in 3 cell lines using normal bone and osteoblasts as controls. Of these candidate fusions, 15 gave the predicted product sizes using breakpoint-spanning PCR, and Sanger sequencing confirmed the identities of 13 of these, giving a validation rate of 76 % (Table 1). However, certain fusion transcripts were detected by PCR in additional samples that were negative by RNA-Seq, suggesting expression levels that were too low to be detected at the sequence depth used. The *EIF5A-HMGN2* and *EEF1A1-VIM* fusion transcripts were detected and validated in all tested cell lines and both controls, indicating that these fusions were not cancer-specific.

**Table 1:** Validation results for 17 candidate fusion transcripts in the osteosarcoma cell lines

Fusion transcript	Positive by RNA-Seq	Breakpoint PCR validation					Sanger sequencing
		IOR/OS15	IOR/MOS	MHM	normal bone	osteoblast	
*COL1A2-MMP14*	IOR/OS15, IOR/OS18	+	+	+	-	-	+
*FUS-LGMN*	IOR/OS15, MG-63	+	+	-	-	-	+
*DHFR-PABPC1*	IOR/OS15	+	+	-	-	-	+
*PTMA-NPM1*	IOR/OS15	+	+	+	-	-	+
*PMP22-ELOVL5*	IOR/OS15	+	+	-	-	-	+
*CDC5L-BTBD9*	IOR/OS15	+	-	-	-	-	+
*RAI1-CLIC5*	IOR/OS15	+	-	-	-	-	+
*TP53-PPRAD*	IOR/OS15	+	-	-	-	-	+
*MRPL39-MAML3*	IOR/OS15	+	-	-	-	-	+
*PLXNA2-RUNX1*	IOR/OS15	+	-	-	-	-	+
*EIF5A-HMGN2*	IOR/OS15, MG-63, Saos-2	+	+	+	+	+	+
*EEF1A1-VIM*	IOR/OS15, IOR/OS18, IOR/SARG	+	+	+	+	+	+
*EIF2S1-RBM25*	IOR/OS15	+	-	-	-	-	+
*COL1A1-HSP90AB1*	IOR/OS15, MG-63	+	-	+	-	-	-
*SCO1-CLIC5*	IOR/OS15	+	+	+	+	+	-
*CREB3L1-HNRPA*	IOR/OS15	-	-	-	-	-	ND
*PDZRN4-PKM2*	IOR/OS15, IOR/OS18, MHM	-	-	-	-	-	ND

(“+” positively validated, “-” not validated, “ND” not determinable)

The expression levels of the wild-type transcripts involved in fusions were significantly enriched for moderate to high expression (Wilcoxon rank-sum test, p-values < 6.36e-05), suggesting a fusion mechanism associated with high expression. In contrast, the majority of fusion transcripts were expressed at lower level.

All sequencing data are available at the European Nucleotide Archive (ENA) under accession number PRJEB7574 (http://www.ebi.ac.uk/ena/data/view/PRJEB7574).

### Identification of structural variations by whole genome sequencing (WGS)

**Figure 1:**
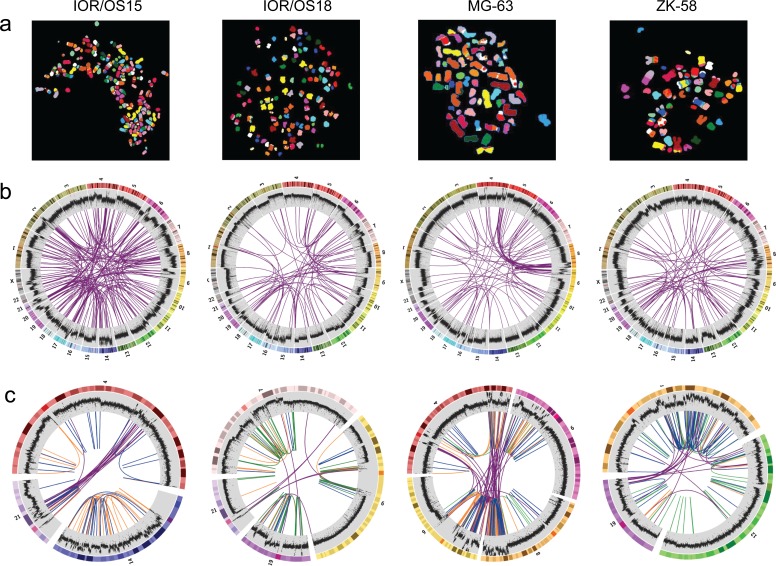
Visualization of the genomic chaos in osteosarcoma **a.** Example single cell multicolor spectral karyotypes for each cell line demonstrating the genomic complexity and high numbers of translocations. **b.** Circos plots showing genome-wide interchromosomal translocations (purple lines) identified by WGS with allele frequencies of ≥10 %. The outermost circles illustrate the chromosome idiograms followed by the plot of the genome coverage (binary logarithmic scale ranging from 2 (log_2_4) to 8 (log_2_256), increasing from center towards periphery, 50K window size). Changes in coverage indicate copy number variations with increase indicating gain and decrease indicating loss. **c.** Circos plots of rearrangement clusters showing chromothripsis-like characteristics of breakpoint distribution within certain chromosomes. Purple lines indicate translocations, orange lines inversions, green lines duplications and blue lines deletions. The coverage plot illustrates high local increase of the copy number, which does not support the copy number neutral chromothripsis model.

All cell lines showed multiple complex chromosomal rearrangements as visualized by spectral karyotyping (SKY, Fig. 1a). Although the resolution is very low, it demonstrated the presence of large numbers of translocations.

**Figure 2:**
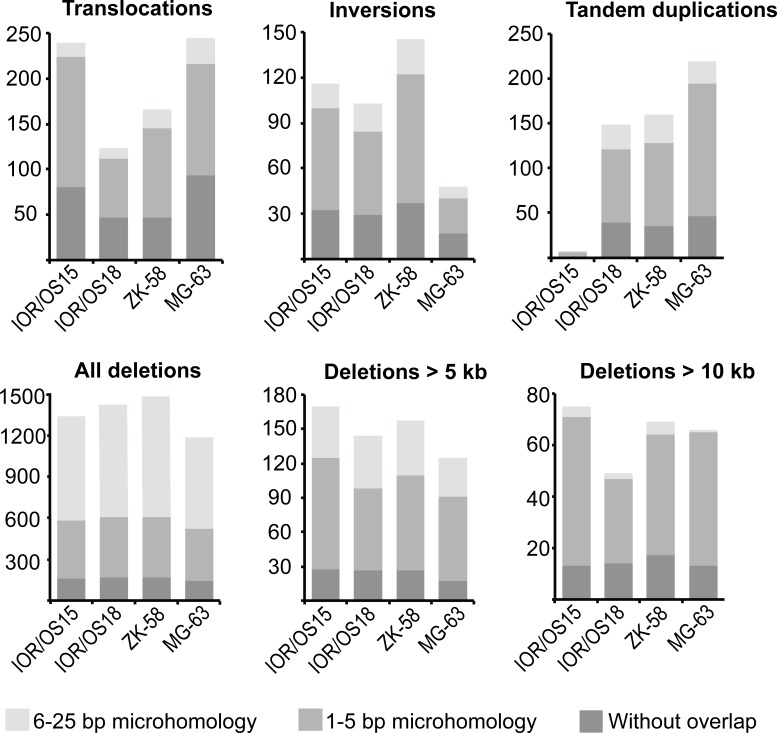
Abundance of the different genomic rearrangement types and their microhomology pattern The rearrangements are grouped by type (translocations, inversions, tandem duplications, deletions) and divided in three categories of overlapping microhomology at the breakpoints: 6-25 bp overlap, 1-5 bp overlap and without overlap. Deletions are further divided by length, showing that smaller deletions (< 5 kb) have a different microhomology pattern with a higher frequency of 6-25 bp long overlapping microhomology. This frequency was clearly reduced for deletions longer than 5 kb and more similar to the other types of rearrangements. The same tendency was found for deletions longer than 10 kb, which showed even more similarity to the microhomology pattern of translocations, inversions and tandem duplications.

The genomes of four cell lines, IOR/OS15, IOR/OS18, MG-63 and ZK-58, were fully sequenced. Approximately 95 % of the more than 1.2 billion paired-end reads from each sample could be mapped to the genome, giving a median coverage of approximately 30x. Upon further filtering, analysis by DELLY showed a high number of deletions, tandem duplications, inversions and interchromosomal translocations (Fig. 2). In general, the frequencies of the different types of rearrangements were similar in the four cell lines, except for a very low number of tandem duplications in IOR/OS15. Large heterogeneity was revealed by allele frequencies of interchromosomal rearrangements from 1 to 50 %. The genomic translocations identified in each sample are given in Supplementary Table S3 and translocations with a frequency above 10 % are presented in Fig. 1b. No translocations were common to all four cell lines, but in all cases *TP53* was affected by rearrangements involving various fusion partners.

All rearrangements were further investigated for microhomology at the breakpoints. We did not detect similarities of more than 25 bp on both sides of the breakpoints, and the rearrangement types were grouped into three categories: 6-25 bp, 1-5 bp, or without microhomology. The ratios of these three categories were similar among translocations, inversions and tandem duplications in all samples, showing that the majority (80-93 %) of breakpoints harbored no more than 5 bp of microhomology. In contrast, deletions were almost 10 times more frequent, and greater than 55 % of these breakpoints harbored microhomologies of 6-25 bp. Interestingly, there was an inverse correlation between the length of deletions and the length of sequence homology at the breakpoint (Fig. 2). The frequency of longer homologies was reduced for deletions longer than 5 kb and showed microhomology patterns more similar to the patterns of translocations, inversions and tandem duplications. The same pattern was observed for deletions longer than 10 kb, which had even more pronounced similarity to microhomology patterns of the other types of genomic rearrangements. The identified microhomology pattern for all rearrangement types differed significantly (p-values < 1.00e-05) from patterns identified by analyzing a random set of 500 sequences derived from the human genome, demonstrating a specific enrichment of 1-5 bp and 6-25 bp microhomologies at the breakpoints.

These findings suggest that somewhat different microhomology mediated DNA repair mechanisms, as i.e. non-homologous end-joining (NHEJ) and microhomology-mediated end-joining (MMEJ) repair, are involved in the generation of the genomic rearrangements and deletions shorter than 5 kb.

Analysis of the genomic distribution of the structural variations showed dense clusters of rearrangements, a characteristic of chromothripsis, in all four cell lines (Fig. 1c). Detailed analysis of the distance between the breakpoints by testing the goodness-of-fit using the null model of random breakpoints following an exponential distribution with the mean of all observed distances [20] was significant (p-values < 0.005) and confirmed the observed feature of chromothripsis. This model was further supported by the randomness of the orientation of DNA fragment joins. The randomness of our data was proven by a Chi-square test for the given probability (p-values > 0.9). However, while chromothripsis is expected to be associated with loss or a neutral copy number [21, 22], we observed more than 6-fold increased coverage compared to the median in these regions. The extreme copy number changes of these cell lines have previously been described using SNP-CGH [3].

### Comparison of RNA-Seq and WGS

For four of the cell lines (IOR/OS15, IOR/OS18, MG-63 and ZK-58) both the whole genome and the transcriptome were sequenced. Surprisingly, for the 182 different fusion transcripts identified in these four cell lines, corresponding genome fusions could only be found for 19. Reduced detection stringency did not improve this rate. Eleven of 93 fusion transcripts showed underlying genomic rearrangements in IOR/OS15, 6 of 101 transcripts in MG-63, 2 of 115 in ZK-58 and none of the 160 fusion transcripts in IOR/OS18 (Table 2). Among the 19 expressed fusion genes, *TP53* was observed three times (IOR/OS15, 2xMG-63). Eight of the 19 fusion transcripts were caused by intrachromosomal rearrangements (deletions or inversions) (translocations). The remaining rearrangements were interchromosomal. Six of the 13 fusion transcripts confirmed by breakpoint PCR at the RNA level (Table 1) could not be confirmed at the genomic level. Even manual inspection of the raw data did not reveal any evidence of underlying genomic rearrangements. Among them were *EIF5A-HMGN2* and *EEF1A1-VIM,* which were also observed in normal samples. One of the fusion transcripts, *SCO1-CLIC5*, for which we were unable to design sufficiently good primers to confirm by breakpoint-spanning PCR, was however unequivocally confirmed by the full genome sequence, with genomic breakpoints that fit perfectly with the identified fusion transcript.

**Table 2:** Genome rearrangements resulting in fusion transcripts identified in three cell lines

Sample	Gene fusion	Rearrangement type
IOR/OS15	*PMP22-ELOVL5*	Translocation
IOR/OS15	*GCC2-CCDC73*	Translocation
IOR/OS15	*RAI1-CLIC5*	Translocation
IOR/OS15	*TP53-PPRAD*	Translocation
IOR/OS15	*MRPL39-MAML3*	Translocation and deletion
IOR/OS15	*PLXNA2-RUNX1*	Translocation
IOR/OS15	*SCO1-CLIC5*	Translocation
IOR/OS15	*EIF2S1-RBM25*	Deletion
IOR/OS15	*BAZ1A-NUMB*	Deletion
IOR/OS15	*CDC5L-BTBD9*	Inversion
IOR/OS15	*MRPL39-NRIP1*	Inversion
MG-63	*DNER-ELL2*	Translocation
MG-63	*CLIP4-EPHB4*	Translocation
MG-63	TP53-VAV1*	Translocation
MG-63	TP53-EMR1*	Translocation
MG-63	*MROH1-PARP10*	Inversion
MG-63	*BNC2-MTAP*	Inversion
ZK-58	*CREBBP-TFAP4*	Deletion
ZK-58	*GDPD5-MAP6*	Deletion

* One genomic translocation (*TP53-VAV1*) causing two different fusion transcripts involving the gene at the breakpoint (*VAV1*) and the gene next to it (*EMR1*).

Correlating the genomic localization of interchromosomal rearrangements relative to genes and their expression gave similar distributions in all four cell lines. Approximately 40 % of the human genome represents intragenic regions (genomic coordinates between start and end of all genes annotated in Homo sapiens GRCh37.74.gft). As 43-61 % of the breakpoints for the different cell lines fall into these regions, there apparently was no significant enrichment. Of the intragenic breakpoints, 15-22 % were located in genes that were not expressed, 22-37 % showed overlap with one expressed gene, and for only 3-10 % both sides of the breakpoints were located in expressed genes (Supplementary Fig. S1). Only 0-2 % resulted in a fusion transcript. Overall, the huge number of chromosomal rearrangements caused only very few fusion transcripts and accordingly the majority of fusion transcripts seem to be generated by other mechanisms.

### *PMP22-ELOVL5* fusion

**Figure 3:**
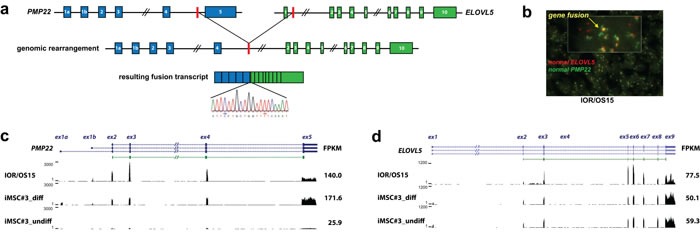
*PMP22-ELOVL5* gene fusion **a.** Schematic overview of the location of the genomic breakpoint and the resulting fusion transcript identified in the cell line IOR/OS15. Validation of the fusion transcript by Sanger sequencing confirmed the breakpoint as illustrated in the sequence chromatogram. **b.** Double-fusion FISH picture of IOR/OS15 in tissue microarray (cell pellets). Green and red probes identify *PMP22* and *ELOVL5,* respectively. Colocalized signals are visible in yellow and confirm the gene fusion at the genome level. Further, normalized expression patterns of *PMP22* **c.** and *ELOVL5* **d.** are shown for IOR/OS15, differentiated and undifferentiated iMSC#3 with the corresponding FPKM (Fragments Per Kilobase of exon per Million fragments mapped) calculated using Cufflinks. For IOR/OS15 a reduced expression of *PMP22* exon 5 and *ELOVL5* exon 1 is visible, caused by the loss of these exons in the fusion transcript.

Fusion genes confirmed at both the RNA and DNA levels were further investigated by fusion-specific TaqMan expression assays in an osteosarcoma panel containing 21 cell lines (including the 11 cell lines used for RNA-seq), 19 patient-derived xenografts and 9 clinical samples. One fusion transcript, *PMP22-ELOVL5,* was recurrent, detected in four cell lines (IOR/OS15, IOR/MOS, MHM, U-2 OS), one xenograft (OKTx), and two patient samples (OS29 and OS83). As the breakpoints for this fusion are located in long introns (intron 4 of *PMP22*, length: 8.4 kb; intron 1 of *ELOVL5*, length: 53 kb), the detailed genomic position could only be determined for IOR/OS15, for which WGS data was available. The expression level of the *PMP22-ELOVL5* fusion transcript was highest in IOR/OS15, while more modestly expressed in the additional samples. Using 5′- and 3′- RACE, we confirmed both transcript ends of the *PMP22*- *ELOVL5* fusion. The products had the expected sizes and the breakpoint was confirmed by Sanger sequencing. The transcript starts at the alternative start site of *PMP22* at exon 1b and continues until exon 4, which is fused to exon 2 of *ELOVL5* and includes the remaining exons (Fig. 3a). The final exon of *PMP22* is lost in the fusion, as well as the non-coding first exon of *ELOVL5*. The resulting protein sequence contains the first 106 of 160 amino acids of PMP22, an additional amino acid (Phe) generated at the fusion, and the complete in frame protein sequence of ELOVL5. The rearrangement was further investigated using fluorescent *in situ* hybridization (FISH) of bacterial artificial chromosomes (BACs) covering the two partner genes on a tissue array of 20 osteosarcoma cell lines (including the ones analyzed by NGS). Abundant co-localization of signals was observed in IOR/OS15, validating the fusion (Fig. 3b). Additional cell lines that were positive for qPCR showed co-localization of the signals in only a fraction of the cells.

Osteosarcomas are thought to originate from a mesenchymal stem cell similar to the ones found in bone marrow [23]. When an immortalized cell line of this type (iMSC#3 [24, 25]) was induced to osteogenic differentiation (Fig. 3c), *PMP22* expression increased to a level higher than was observed in any of the osteosarcoma lines, suggesting a role in osteoblast differentiation (Supplementary Table S4). The cell line IOR/OS15, harboring the *PMP22-ELOVL5* gene fusion and a genomic amplification of the whole *PMP22* locus, was higher expressed only for the part of the transcript involved in the fusion (Fig. 3c). Despite the amplification, the expression level of exon 5, specific to wild-type *PMP22* (lost in the gene fusion), was lower, and similar to that in the differentiated iMSC#3. The same tendency was observed for *ELOVL5:* there was little change when iMSC#3 cells were differentiated, and only IOR/OS15 exhibited higher expression (Fig. 3d, Supplementary Table S4).

The presence of the fusion gene was further investigated in 25 WGS data sets of osteosarcoma tumors (part of the International Cancer Genome Consortium, Bone cancer project). *PMP22* was translocated in one of these samples, but the breakpoint in intron 3 was fused to an intergenic region of chromosome 5. Thus, this translocation t(17;5)(p12;p15.1) appears to result in a truncation of the *PMP22* transcript rather than a *bona fide* fusion transcript. Because of a lack of RNA material for these samples, the presence of the fusion RNA could not be investigated.

Summarizing the findings in the two sample sets (49 samples investigated at RNA and 25 samples at DNA level), *PMP22-ELOVL5* occurred at a frequency of 9 % (7 of 74) and is the first recurrent fusion transcript reported in osteosarcoma.

### Aberrations and activity of *TP53*

**Figure 4:**
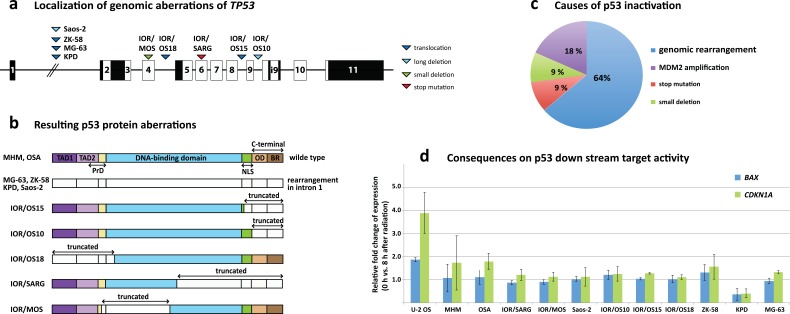
*TP53* aberrations in osteosarcoma cell lines **a.** Genomic localization of aberrations across *TP53* with triangles demonstrating the position of the aberrations for the different cell lines and the aberration type (dark blue = translocation, light blue = long deletion, green = small deletion, red = stop mutation). **b.** Visualization of the consequences of the *TP53* aberrations at the protein level. The different protein domains are indicated (TAD = transactivation domain, NLS = nuclear localization signal, OD = oligomerization domain, BR = basic region). Uncolored regions of the protein represent truncated parts. **c.** The distribution of the various causes of p53 inactivation clearly shows that rearrangements in *TP53* are the major factor with 64 % followed by *MDM2* amplifications, small deletions and mutations. **d.** Results of the radiation-induced DNA damage assay showing the fold change of *BAX* and *CDKN1A* expression 8 hours after induction. U-2 OS is known to harbor wild-type p53 and was used as control.

Another prominent finding was the frequent rearrangements of *TP53*. Four of eleven sequenced cell lines (KPD, MG-63, Saos-2, IOR/OS15) expressed fusion transcripts involving *TP53*. In MG-63, KPD and Saos-2, the fusion transcripts contained only the first (non-coding) exon, which was fused to the second exon of *VAV1,* the sixth exon of the *DDX39B* and the first exon of *SAT2,* respectively. The translocation in MG-63 seemed to result in two different fusion transcripts, one involving the gene at the breakpoint (*VAV1*) and another transcript involving the gene next to it (*EMR1*). No further rearrangement could be found that could explain the *TP53-EMR1* fusion transcript, suggesting some kind of read-through followed by splicing out of the *VAV1* sequences. The transcript found in IOR/OS15 contained exons 1 to 8 (amino acids 1-306) of *TP53* fused to the second exon of *PPRAD*. The loss of the last 87 amino acids (aa) from the IOR/OS15 fusion removes most of the nuclear localization signal and C-terminus of p53. In Saos-2, the fusion transcript is the result of a long deletion after exon 1. Further analysis of the *TP53* loci in the cell lines at the DNA and RNA level revealed aberrations in 5 additional cell lines. IOR/OS10 contained a truncated transcript that ended after exon 9 (aa 331) and thereby had lost the complete C-terminal region, including the oligomerization domain and the basic region. ZK-58 and IOR/OS18 exhibited genomic translocations (t(5;17)(q13.2;p13.1) and t(14;17)(q32.2;p13.1), respectively) that generated truncated *TP53* mRNAs confirmed at the RNA level. The resulting transcript for ZK-58 included only the first exon and for IOR/OS18, exon 5-11 (aa 126-393), which led to the loss of both transactivation domains (TAD1 and TAD2) and the first 25 aa of the DNA-binding domain. In IOR/SARG, we identified a homozygous stop mutation at aa 205, resulting in the loss of the last 87 aa of the DNA-binding domain, the nuclear localization signal and the C-terminus. The previously described deletion at bp 249-572 in IOR/MOS [26], which resulted in a deletion affecting TAD2 and the DNA binding domain, was confirmed. Thus, 9 of 11 osteosarcoma cell lines showed fused or truncated *TP53* mRNAs that compromised the p53 protein structure (Fig. 4a, 4b), with a rearrangement hot spot in intron 1 (4 samples). Wild-type *TP53* mRNA was detected only in IOR/MOS, MHM and OSA. In the latter two cell lines, the pathway is inactivated by *MDM2* amplification, leading to 10-fold increased expression of *MDM2* [26, 27]. IOR/MOS has low-level expression of *TP53* from an apparently normal allele, and we do not know whether this causes haploinsufficiency or if another component of the pathway also is affected.

To determine the activity of the p53 pathway, all cell lines were subjected to radiation-induced DNA damage, and the induction of two major targets downstream of p53 was determined – *CDKN1A* (p21) and *BAX*. The cell line U-2 OS, which is a human osteosarcoma cell line expressing wild-type p53 and RB1, but lacking p16 [28, 29], was used as the control. The comparison of expression levels before and 8 hours after radiation-induced DNA damage showed induction of *CDKN1A* and *BAX* in the control U-2 OS but no significant increase in any of the other tested cell lines (Fig. 4d). A slight increase was detected in MHM and OSA, the two cell lines harboring wild type p53 but also *MDM2* amplification. This demonstrates loss-of function of p53 in all osteosarcoma cell lines (except U-2 OS), caused in 7 cases by genomic rearrangements affecting *TP53,* in 2 cases by *MDM2* amplification and in 1 case by a mutation and a small deletion (Fig. 4c).

Detailed investigation of the *TP53* locus in the WGS data from 25 osteosarcoma tumors revealed rearrangements that directly affected *TP53* in ten samples (Table 3), also with a hotspot in intron 1 (5 samples) and a second hotspot in intron 9 (2 samples). Somatic *TP53* mutations/indels were found in six samples and high copy number amplification of *MDM2* in two samples. The rearrangements caused the loss of critical parts of the protein, probably resulting in loss of function. Certain translocations in the *TP53* region involved other genes (*WDR25*, *SOX5*, *PNKP*, *ZNF787*, *EIF3L, ACAN, PIGU*), but RNA was not available, so fusion transcripts could not be investigated. We identified one sample with a 10 bp deletion removing the junction of exon 5 and the following intron that would affect splicing and lead to a change in the reading frame. Furthermore, 5 samples had severe somatic missense mutations in *TP53*, which are recorded in the COSMIC database. Three of the mutations (p.C238F, p.D281E, p.R273L) cause a loss of function of p53 [30]. Less is known about the impact of the remaining two mutations (p.I255T, p.C275Y). For all tumor samples in this study with aberrant *TP53*, the wild-type *TP53* allele frequencies varied between 15 % and 80 % with a median of 30 %, which probably mostly originated from non-aberrant cells in the tumor stroma. In addition, 2 of the 9 samples without *TP53* aberrations contained high copy number amplification of *MDM2* (DNA Copy Number of 20 and 60). Altogether, 18 of the 25 tumor samples harbored somatic aberrations that affect the p53 pathway. Two of the patients without detectable somatic aberrations of *TP53* turned out to have germline aberrations (not shown), thus leaving only 5 of 25 without aberrations of *TP53*.

**Table 3:** Overview of *TP53* aberrations and *MDM2* amplification identified in the 25 osteosarcoma tumors

Gene/Sample	Type of aberration	Position	Consequence	Aberr. allele frequency	WT allele frequency
***TP53***					
PD7507a	Del (7.5 Mb)	Intron 9	TSS-exon i9 deleted	40%	20%
	Trans	Intron 9	Loss of exon i9-3′UTR	40%	
PD7513a	Trans	Intron 1	Loss of exon2-3′UTR	40%	25%
	Trans	Intron 1	Loss of TSS and exon1	35%	
PD7194a	Inv (50.3 Mb)	Intron 1	Loss of exon2-3′UTR	40%	30%
	Trans	1.8 Mb upstream *TP53* TSS	Gene loss (unbalanced translocation)	30%	
PD7197a	Small del (10 bp)	Exon5/intron5	Splice site disruption	55%	45%
PD13491a	Del (416kb)	364 kb upstream - 31 kb downstream *TP53* 3′ UTR	Gene loss	70%	30%
PD13492a	Trans	Intron 1	Loss of exon2-3′UTR	55%	15%
	Trans	Intron 1	Loss of TSS and exon1	30%	
PD13493a	Del (72 kb)	50 kb upstream - 3 kb downstream *TP53*	Gene loss	60%	40%
PD13496a	Trans	76 kb downstream *TP53*	Gene loss	50%	30%
	Trans	2 kb upstream *TP53*	Gene loss	20%	
PD7193	Del (93 kb)	Intron 1	Loss of exon2-3′UTR	40%	30%
	Trans	Intron 1	Loss of exon2-3′UTR	30%	
PD13485a	Del (9 kb)	Intron 9	Loss of exon i9-3′UTR	20%	80%
PD13486a	Trans	Intron 1	Loss of exon2-3′UTR	75%	25%
PD13484a	SNV	c.843C>A	p.D281E	32%	68%
PD13487a	SNV	c.713G>T	p.C238F	82%	18%
PD13488a	SNV	c.764T>C	p.I255T	62%	38%
PD7196a	SNV	c.818G>T	p.R273L	47%	53%
PD13490a	SNV	c.824G>A	p.C275Y	72%	28%
***MDM2***					
PD7508a	High level Ampl	chr12 q15	Total CN of 20	-	10 fold increased
PD7510a	High level Ampl	chr12 q15	Total CN of 60	-	30 fold increased

(WT= wild-type, Aberr= aberrant, Ampl= amplification, Del= deletion, Trans= translocation, Inv= inversion, SNV= single nucleotide variation, CN= copy number)

### Rearrangements of *RB1*

Further evaluation of findings made in cell lines revealed that *RB1* was recurrently affected by rearrangements in 3 out of 11 cell lines and in 5 out of the 25 tumor samples. Similar to *TP53,* rearrangements varied and did not generate a specific recurrent gene fusion but rather a truncation or deletion of the transcripts.

The cell lines IOR/OS10 and IOR/SARG exhibited almost undetectable expression of *RB1*. The SNP-CGH data from a previous study [3] of these cell lines confirmed a heterozygous deletion in IOR/OS10 in the *RB1* region and a copy number increase at the beginning of the gene, creating a breakpoint in IOR/SARG. In Saos-2 the transcript was truncated after exon 20*,* with evidence of a 7.5 Mb deletion in the RNA-Seq data. In the tumor cohort we identified 5 samples harboring rearrangements in or around the *RB1* locus, with 4 samples containing translocations (2 in intron 2, 1 in intron 7, 1 in intron 21) and one sample with a 2.7 Mb deletion covering the whole gene. All patient samples with *RB1* aberrations also showed *TP53* aberrations.

### *MTAP* rearrangements with *CDKN2A* co-deletions

An *MTAP-BCN2* fusion transcript was identified in the osteosarcoma cell line MG-63 by RNA-Seq and confirmed as a genomic inversion on chromosome 9. The specific fusion transcript was not recurrent, but further investigation revealed two more cell lines containing *MTAP* rearrangements. The cell lines IOR/OS15 and IOR/OS18 showed no detectable expression, which was caused by a genomic 2.95 Mb deletion in IOR/OS18 and unbalanced translocation 35 kb upstream in IOR/OS15. In all three cases, the neighboring *CDKN2A* locus was co-deleted. In addition, in the patient cohort, we identified rearrangements of *MTAP* in three of the 25 samples. One sample contained three different unbalanced translocations in the *MTAP* region, whereas the other two contained different deletions, including a complete deletion of the gene and a deletion in intron 4 leading to a truncation from exon 5 and upstream. As in the cell lines, the *CDKN2A* locus was co-affected in all three cases. Further two of the patient samples with *MTAP/CDKN2A* aberrations did not show any *TP53, RB1* or *MDM2* aberration.

## DISCUSSION

Combining extensive information about structural genomic aberrations and their consequences at the RNA level revealed highly frequent rearrangements affecting the tumor suppressor genes *TP53*, *RB1* and *CDKN2A,* and for the first time a recurrent gene fusion, involving *PMP22* and *ELOVL5,* and extensive transcript fusion without genomic evidence as a characteristic of osteosarcoma.

At RNA level the validation rate of 76 % of the 17 candidate fusion transcripts tested here suggests a high likelihood for the remaining candidates to be valid, although the majority of fusion transcripts could not be confirmed at the genome level. Even for six of the extensively validated RNA fusions we could not find any evidence of an underlying genomic rearrangement. Although it is possible that this in certain cases was caused by the moderate coverage of WGS combined with aneuploidy, it seems unlikely that this would explain the discrepancy in all cases. Thus, biological mechanisms, such as trans-splicing or errors of the transcription processes, are more likely to explain those fusion transcripts. Such chimeric transcripts are found in normal human cells including stem cells and are shown to play important roles, i.e. in human embryonic stem cell pluripotency [31, 32]. In prostate cancer [33] and colorectal cancer [34] a high number of fusion transcripts were identified also with subsets detected in benign tissue. Further, in a subset of prostate cancer a highly induced, androgen-regulated fusion transcript (*SLC45A3-ELK4*) was identified, mainly occurring through a mechanism other than genomic rearrangements [35]. These studies also showed that genes involved in fusion transcripts were highly tissue specific and significantly enriched for high expression, whereas the fusion transcripts themselves were modestly expressed, as was also observed for most of the fusion transcripts in our study. Further, we confirmed the expression of *EIF5A-HMGN2* and *EEF1A1-VIM* fusion transcripts with exactly the same chimeric structure in normal bone and primary osteoblasts, suggesting a normal precise mechanism independent of gene rearrangements. As we could not detect expression of these transcripts in other tissues, they appear to be bone-specific. In general, our results suggest that the majority of fusion transcripts in osteosarcoma are generated at the RNA level, and further studies are necessary to determine the mechanisms involved and their biological impact in both normal cells and cancer. The hyperactive production of fusion transcripts thus appears to be a phenotype of osteosarcoma and recurrent fusion transcripts represent putative biomarkers.

WGS confirmed the complexity and high level of heterogeneity of osteosarcoma genomes. We found strong evidence for the involvement of non-homologous end-joining (NHEJ) and microhomology-mediated end-joining (MMEJ) DNA repair in the generation of the structural aberrations of the genome. Our results further indicate that MMEJ was primarily involved in the generation of deletions shorter than 5 kb. This result is consistent with earlier studies showing that MMEJ repair of double-strand breaks (DSB) most often results in deletions [36].

The clustering of genome rearrangements observed was similar to what has been described for chromothripsis [20]. However, in contrast to classical chromothripsis, the associated DNA copy number increase suggests the involvement of microhomology mediated break-induced replication (MMBIR). This process can generate chromothripsis-like complex genomic rearrangements that include amplification through increased replication at DSBs [37]. As this mechanism and the increased level of MMEJ can explain the majority of rearrangements, and both are connected to replication forks collapsed in the S-phase, this process is likely to be a major factor in osteosarcoma. Further investigation of the cell cycle and replication mechanisms could provide additional information on how MMBIR and MMEJ are activated in osteosarcoma and whether the observed absence of p53-induced DNA damage response is required for such activation. Overexpression of exogenous wild-type p53 or its reactivation in osteosarcoma cell lines with amplification of *MDM2* causes cell cycle arrest, indicating that the conditions for growth arrest are present, but the p53 response is lacking [27, 28]. This defect most likely leads to a further increase of DSBs and the need for MMBIR and MMEJ to repair them, leading again to an increased number of rearrangements. This scenario would also explain the high degree of heterogeneity in osteosarcomas, as these continuous processes would generate new rearrangements at each cell division.

In total, 19 fusion transcripts caused by genomic rearrangements were identified in three of the four cell lines analyzed by WGS and only one, *PMP22-ELOVL5,* could be further validated in multiple samples. The recurrent *PMP22*-*ELOVL5* fusion transcript was detected in four cell lines, one xenograft and two patient samples. This specific fusion was not observed in any of the 25 tumors analyzed by WGS, but one contained a translocation of *PMP22* but fused to an intergenic region. Due to the lack of RNA from the patient samples, the presence of a fusion transcript could not be investigated. Observed in 7 independent samples, this is the first recurrent translocation to be identified in osteosarcoma. The *PMP22* gene encodes a 22 kDa glycoprotein member of an extended family of tetraspan membrane proteins that play important roles in myelin membrane formation and, when mutated, are responsible for a set of inherited peripheral neuropathies, including Charcot-Marie-Tooth disease [38]. Further studies have demonstrated that PMP22 can regulate cell death, shape, migration, and its involvement in the p53-dependent apoptotic response in several cell types [39, 40]. Inhibition of the p53-dependent apoptotic response by growth arrest attenuated *PMP22* expression*,* whereas overexpression of *PMP22* could trigger an apoptotic response. Thus, the general inactivation of p53 described here could explain the reported lack of induction of *PMP22* expression in growth-arrested osteosarcoma cell lines, including MG-63 [41]. Mutations within the transmembrane domains of PMP22 cause impaired transport to the cell surface and intracellular accumulation [42]. These mutants were also unable to induce cell death. The resulting *PMP22-ELOVL5* fusion protein has lost the last 54 amino acids of PMP22, which code for parts of the third and the complete fourth transmembrane domain, possibly resulting in loss of tumor suppressor activity. However, the relationships of *PMP22* expression level to prognosis and the outcome of different cancer types are contradictory [43-45].

*ELOVL5* is a fatty acid elongase located in the plasma membrane. Very little is known about its possible association with cancer, apart from a study reporting down-regulation of *ELOVL5* in prostate cancer tissue [46]. However, this was not the case in our osteosarcoma cell lines.

The most striking finding in our study was the high frequency of rearrangements affecting *TP53*. Inactivating genomic rearrangements were identified in 7 of 11 cell lines and 10 of 25 patient biopsies. *TP53* intragenic rearrangements represent a new mechanism of p53 inactivation and are very frequent in osteosarcoma [17, 19]. Two cell lines (IOR/MOS, IOR/SARG) and 5 tumors were affected by previously described point mutations that caused partial loss of function of p53 [30]. Two cell lines (MHM, OSA) and two tumor samples harboring wild type p53 showed inactivation of the p53 pathway by *MDM2* gene amplification [47]. Our radiation-induced DNA damage assay verified that p53 activity is compromised in all examined cell lines except for U-2 OS, which was used as a wild-type *TP53* control. Although this result may not be surprising for a tumor type with such chaotic genomes, we cannot exclude that this strikingly high prevalence in cell lines is selected for during *in vitro* culture. The analysis of 25 full-genome sequences from patient biopsies revealed a similar pattern of *TP53* rearrangement in 40 % of the samples. Altogether, our results show that p53 inactivation in osteosarcomas by rearrangement (40 %), mutation/indels (24 %) or *MDM2* amplification (8 %) is much more frequent than previously reported [12, 48]. Additionally two samples showed germ line *TP53* aberrations (not shown). This confirms the key role of p53 in osteosarcoma, consistent with its critical role in Li-Fraumeni syndrome and osteoblast biology [49]. The frequent structural rearrangements of *TP53* with a hotspot in intron 1 (5 patient samples, 4 cell lines) were also found in two recent WGS studies of osteosarcomas [17, 18]. In addition, intron 9 was also found to be frequently affected by rearrangements (2 patient samples, 1 cell line) representing a second hotspot. In contrast to the previous study, 5 of our osteosarcoma patient samples (20 %) did not show any aberration of *TP53, RB1* or *MDM2*. But two of these samples showed *MTAP* rearrangement with *CDKN2A* co-deletion. *CDKN2A* codes for an inhibitor of CDK4, already known to be critical in the regulation of the activity of retinoblastoma protein (RB1) [50], and also encodes p14^ARF^, which targets MDM2 for degradation [51], probably causing an inhibition of the p53 and RB1 pathways in these two cases.

*MTAP* encodes methylthioadenosine phosphorylase, essential for adenine and methionine synthesis, and was deleted in 38 % of osteosarcoma tumors [52]. Mutations in *MTAP* may result in diaphyseal medullary stenosis with malignant fibrous histiocytoma (DMS-MFH), an autosomal-dominant syndrome characterized by bone dysplasia, myopathy, and bone cancer [53]. As found in our study, the tumor suppressor gene *CDKN2A* was always co-affected, and it is likely that *CDKN2A* is the main target.

Further, the *RB1* locus was frequently affected (20 %), RB1 being essential for the regulation of cell cycle progression at the G1/S checkpoint. In previous studies, 25-35 % of osteosarcomas showed mutations or rearrangements of *RB1* [17, 54].

The molecular dissection of osteosarcoma revealed an extraordinary degree of genome rearrangement harboring highly recurrent cancer specific aberrations, the involvement of microhomology mediated repair mechanism and extensive transcript fusions, apparently caused by a trans-splicing phenotype. Furthermore, we identified systematic inactivation of the p53 pathway by rearrangements of *TP53*. Unscrambling the genomic chaos of osteosarcoma at base-pair resolution provided new insights in mechanisms involved in osteosarcoma development and may contribute to develop new diagnostic and therapeutic strategies.

## MATERIALS AND METHODS

### Samples

Tumor samples were collected at the Department of Pathology, Oslo University Hospital, and diagnosed according to the current World Health Organization classification [55]. The project and informed consent were approved by the Ethical committee of Southern Norway (Project S-06133). Data for all tumor samples are summarized in Supplementary Table S1. Nineteen osteosarcoma cell lines from the EuroBoNeT panel [26] plus G-292 (ATCC, Manassas, VA, USA) and Cal72 (University College London, London, UK) were also analyzed, 11 of which were included in the NGS analysis. All cell lines were mycoplasma negative and authenticated by STR DNA profiling using Powerplex 16 (Promega, Madison, WI, USA); the data were validated using the known profiles of the EuroBoNeT cell bank and ATCC.

Normal bone samples were obtained from amputations of osteosarcoma cancer patients, collected as far away as possible from the tumor site and exhibiting normal DNA copy numbers. Two other normal bone samples were purchased from Capital Biosciences (Gaithersburg, MD, USA). Two normal osteoblast samples were from human calvaria (ScienCell Research Laboratories, San Diego, CA, USA), and two were from femur and tibia (Cambrex BioScience, Maryland, USA). Data for all normal samples are given in Supplementary Table S4. For RNA-Seq analysis, a mesenchymal stromal cell (MSC) line, undifferentiated and differentiated into osteoblasts, was used as a normal control [24].

The sample set of the 25 osteosarcoma tumor samples is a part of the Bone cancer project of the International Cancer Genome Consortium (IGCG) and sequencing data were generated at the Wellcome Trust Sanger Institute (Hinxton, UK).

### Library preparation and sequencing

Genomic DNA isolation was conducted using Wizard Genomic DNA Purification Kit (Promega), and total RNA was isolated using the miRNeasy Mini Kit from QIAGEN (Germany). Total RNA was assessed for quality on an Agilent Technologies 2100 Bioanalyzer (Agilent Technologies Inc., CA, USA).

RNA sequencing libraries were created using the Illumina TruSeq RNA Sample Preparation Kit v2 (Illumina Inc., CA, USA), and 1 μg high-quality total RNA starting material was processed according to the manufacturer's instructions.

The resulting libraries were sequenced on the Genome Analyzer IIx using TruSeq SBS Kit v5 to perform paired-end runs with 75 bp sequence length (2 × 75 bp). Real-time analysis and base calling were conducted by Illumina's software packages SCS2.8/RTA1.8 and Off-line Basecaller v1.8.

DNA library construction and whole genome sequencing (WGS) of four cell lines were conducted at the Beijing Genomics Institute (www.genomics.cn) using paired-end sequencing, 2x 100 bp read length and 500 bp fragment length on the Illumina HiSeq platform.

The sequencing data generated in this project were submitted to the European Nucleotide Archive (ENA) and are available under accession number PRJEB7574.

### RNA-Seq data analysis

RNA-Seq data were analyzed using the deFuse algorithm [56] and TRUP [57], both of which are computational methods for fusion RNA discovery. Applying these methods, paired-end reads were aligned to the *Homo sapiens* GRC37.58 assembly to detect fusion events by resolving ambiguous discordant alignments and identifying the most likely assignment of reads spanning a breakpoint (spanning reads) and reads harboring a breakpoint (breakpoint-covering reads). Both methods additionally combine the read-pair analysis with *de novo* assembly. The predominantly overlapping results from both tools were combined for further analysis.

Possible mapping artifacts, ribosomal contamination, and normal trans-splicing events were removed by filtering out any potential candidate fusions detected in the 16 normal tissues available from Illumina's BodyMap2.0 (Gary Schroth, Illumina), as well as candidates with very high repetition or low complexity sequence 200 bp around the breakpoint. Finally, only fusion transcripts with at least 8 breakpoint-spanning and 3 breakpoint-covering (split) reads were reported.

Transcript expression analysis was conducted using the TopHat/Cufflinks/Cuffcompare pipeline [58], and differences in expression values were determined by the Wilcoxon rank-sum test, assuming the observations from the different groups to be independent of each other and equal under the null hypothesis.

### DNA-Seq data analysis

The DNA sequencing data were mapped to the human genome build hg19 (http://hgdownload.cse.ucsc.edu/goldenPath/hg19/bigZips/) using BWA (v0.6.1) and genomic structural variants at single base pair resolution by integrated paired-end and split-read analysis by DELLY [59], requiring at least 8 spanning reads and 3 breakpoint-covering reads. Overlapping homologies at the breakpoint junctions, also identified using DELLY, were divided into three groups: no overlap, 1-5 bp and 6-25 bp long microhomology. To test for randomness, the distributions among the groups for all types of rearrangements were compared to the distributions of microhomologies identified by analyzing 500 random sequences that represent the average genome (described by Vissers *et al.*) [60], using Fisher's exact test for count data with simulated p-values (based on 1.00e+05 replicates).

The allele frequency of interchromosomal translocations was calculated by comparing the number of concordant reads and number of breakpoints spanning and covering reads in a 500-bp window around the breakpoint. Further statistical analysis on the clustering of breakpoints and the randomness of aberration types (deletion, insertion, tandem duplication, translocation) was conducted in the statistical programming language R following the recommendations of Korbel and Campbell in “Criteria for Inference of Chromothripsis in Cancer Genomes” [20].

### Validation and screening

Primers for breakpoint-spanning PCR were placed approximately 100 bp upstream and downstream from the breakpoint and designed by the NCBI primer-designing tool (http://www.ncbi.nlm.nih.gov/tools/primer-blast/). If agarose gel electrophoresis showed a PCR product in the expected size range, the band was isolated and analyzed by Sanger sequencing at Eurofins MWG (Eurofins Genomics, Ebersberg, Germany). Detection of expression was conducted using TaqMan Gene expression assays (Life Technologies, CA, USA), manually designed using PrimerExpress v3 with probes located over the breakpoint of the fusion transcript to be able to distinguish the normal transcripts and specifically measure expression of the fusions. The 5′- and 3′- ends of individual fusion transcripts were amplified using a SMARTer 5′- and 3′-RACE PCR kit (Clontech Laboratories, Inc., CA, USA).

### Cytogenetic techniques

FISH (Fluorescence *in situ* hybridization) was used to validate the underlying genomic rearrangements using formalin-fixed paraffin-embedded tissue array slides (tissue microarrays, TMAs) of 20 osteosarcoma cell lines and BACs covering *PMP22* (RP11-47J1) and *ELOVL5* (RP11-142f5). To generate TMAs, each cell line was cultured in 6–8 75 cm^2^ cell culture flasks. Cells were washed with RPMI and scraped out with 1 ml 4% formalin and sedimented by brief centrifugation. The cell pellets were treated for at least 48 hours in formalin. Before pellets were embedded in paraffin, the cells were dehydrated in ethanol (70, 90, 95, and 99%) and xylene. To produce a cell line array of all of the cell lines, punches of 2 mm in diameter of the embedded cells were arranged on one prepared paraffin block (Beecher Instruments, WI). Interphase FISH and spectral karyotyping (SKY) were performed according to previously described protocols by Kresse *et al.* (2012) [3].

### Radiation-induced DNA damage

The cell lines were plated in 6-well plates at 80 % confluence and left to attach overnight. The following day, the cells were irradiated with 5 Gy using an X-ray generator (Faxitron CP160, 160kV, 6.3 mA). The cells were harvested at 0 h and 8 h after irradiation using a cell scraper and ice-cold PBS solution (Lonza). The cells were spun down at 300 × *g* for 5 min and immediately frozen at −80°C. RNA was isolated from the pellets using the RNeasy micro kit (QIAGEN) and reverse-transcribed to cDNA using the SuperScript VILO cDNA synthesis kit (Life Technologies) according to the manufacturer's instructions. For gene expression analysis of *CDKN1A* (assay ID: Hs00355782_m1) and *BAX* (assay ID: Hs00355782_m1), quantitative real-time RT-PCR (qRT-PCR) was performed using TaqMan Gene Expression Assays (Life Technologies). Relative expression was analyzed using the 2^−ΔΔCt^-method [61], using TATA-box binding protein (*TBP*, assay ID Hs99999910_m1) as an endogenous reference for normalization.

## SUPPLEMENTARY MATERIAL FIGURE AND TABLES










