# Supplementary material for: Unscrambling the genomic chaos of osteosarcoma reveals extensive transcript fusion, recurrent rearrangements and frequent novel TP53 aberrations
Source: Oncotarget. 2015 Dec 11;7(5):5273–88. doi: 10.18632/oncotarget.6567 (PMC4868685; doi:10.18632/oncotarget.6567)
Supplement: Supplementary file 1 [file oncotarget-07-5273-s001.pdf]

Unscrambling the genomic chaos of osteosarcoma reveals extensive transcript fusion, recurrent rearrangements and frequent novel TP53 aberrations

Supplementary Material

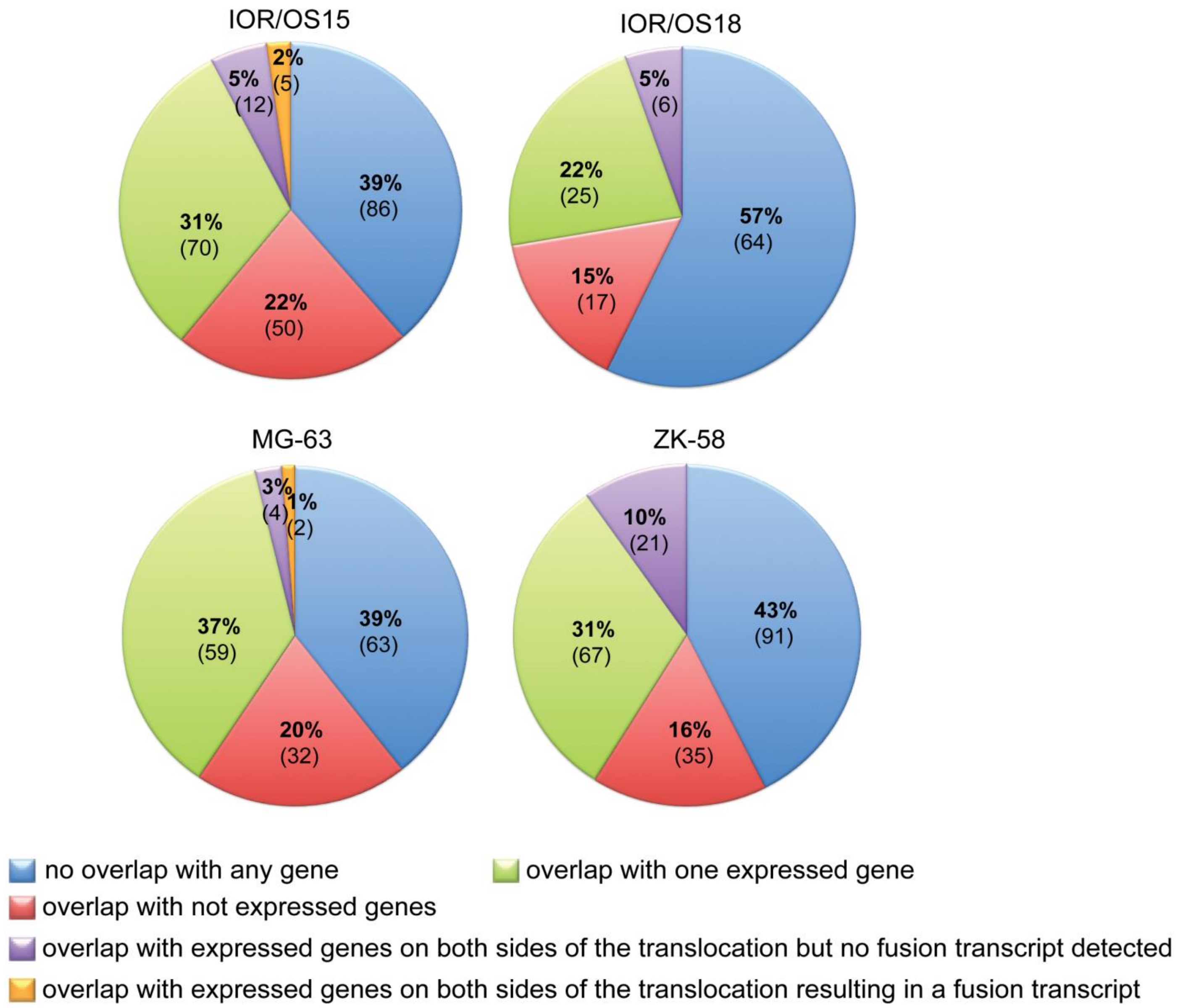

Supplementary Figure1: Distribution of genomic translocations according to their overlap with intragenic regions and gene expression. The translocations are divided into different categories dependent on their overlap with genes and further if the genes are expressed or not. The majority of translocations is not located in intragenic regions (absolute numbers of translocation given in parentheses). Even if both ends of the translocations are located in genes, only for a small proportion (3-10 %) these genes are expressed and only very few generate fusion transcripts.
